# Oxygen Variations—Insights into Hypoxia, Hyperoxia and Hyperbaric Hyperoxia—Is the Dose the Clue?

**DOI:** 10.3390/ijms241713472

**Published:** 2023-08-30

**Authors:** Costantino Balestra, Simona Mrakic-Sposta, Fabio Virgili

**Affiliations:** 1 Environmental, Occupational, Aging (Integrative) Physiology Laboratory, Haute Ecole Bruxelles-Brabant (HE2B), 1160 Brussels, Belgium; 2 Anatomical Research and Clinical Studies, Vrije Universiteit Brussels (VUB), 1090 Brussels, Belgium; 3 DAN Europe Research Division (Roseto-Brussels), 1160 Brussels, Belgium; 4 Physical Activity Teaching Unit, Motor Sciences Department, Université Libre de Bruxelles (ULB), 1050 Brussels, Belgium; 5 Institute of Clinical Physiology, National Research Council (CNR), 20162 Milan, Italy; simona.mrakicsposta@cnr.it; 6 Interuniversitary Consortium “National Institute for Bio-Structures and Bio-Systems”—I.N.B.B., 00136 Rome, Italy; fvirgili@outlook.it

Molecular oxygen (O_2_) is one of the four most important elements on Earth (alongside carbon, nitrogen and hydrogen); aerobic organisms depend on it to release energy from carbon-based molecules. The concentration of oxygen in the atmosphere is ~20.93–20.95% (209–460 ppm), but this has fluctuated markedly throughout geological history. It stabilized within a habitable range, between ~15% and 35%, which has been maintained from the Cambrian period 540 million years ago until today [1].

The history of the use and the study of oxygen is of great interest, yet we firmly believe that it has not yet reached a final point.

Nowadays, the therapeutic use of oxygen is not only limited to restoring hypoxia, but several newly developed approaches use oxygen not only as a “restoring agent” [2] but also as a potent stimulus [3]. Salvagno et al. based their review on the paradoxical response of the intermittent shift between hyperoxic–normoxic exposure, which was shown to enhance erythropoietin production and raise hemoglobin levels with numerous different potential applications in many fields of therapy as a new strategy for surgical preconditioning aimed at frail patients and prevention of postoperative anemia. They summarize the physiological processes behind the proposed “normobaric oxygen paradox”, focusing on the latest scientific evidence and the potential applications for this strategy [4,5].

The Renaissance physician Paracelsus noted that “Nothing is without poison—the poison is in the dose”. The contemporary interpretation of this statement is that dose and effect move together in a predictably linear fashion, and therefore, lower exposures to a hazardous compound will always generate lower risks. The new data presented in this Special Issue open new perspectives and explore the “linearity” of cellular responses to oxygen doses [1,6,7,8].

Balestra et al. [9] focused on the production of cellular microparticles after one hour of different levels of oxygen exposure in healthy subjects. They analyzed six different oxygen breathing concentrations from hypoxia to hyperbaric hyperoxia (See Figure 1).

Microparticles (MPs) expressing proteins specific to different cells were analyzed, including platelets (CD41), neutrophils (CD66b), endothelial cells (CD146), and microglia (TMEM). Phalloidin binding and thrombospondin-1 (TSP), which are related to neutrophil and platelet activation, were also analyzed. The responses were found to be different and sometimes contrasting. Significant elevations were identified for MPs expressing CD41, CD66b, TMEM, and phalloidin binding in all conditions apart from 1.4 ATA, which elicited significant decreases. Few changes were found for CD146 and TSP [10,11,12,13].

Such results challenge the “paracelsian” view on oxygen. Previous studies have already shown the different magnitudes and speeds of reactions after oxygen exposure of cellular HIF-1 α at various levels. Fratantonio et al. [14] described the activation time trend of oxygen-sensitive transcription factors in human peripheral blood mononuclear cells (PBMCs) obtained from healthy subjects after one hour of exposure to mild (MH), high (HH), and very high (VHH) hyperoxia, corresponding to 30%, 100%, and 140% O_2_, respectively. They confirmed that MH is perceived as a hypoxic stress, characterized by the activation of HIF-1 α and nuclear factor (erythroid-derived 2)-like 2 (NRF2), but not of the Nuclear Factor kappa-light-chain-enhancer of activated B cells (NF-kB). Conversely, HH is associated with a progressive increase in oxidative stress leading to NRF2 and NF-kB activation, accompanied by the synthesis of glutathione (GSH). After VHH, HIF-1 α activation is totally absent and oxidative stress response, accompanied by NF-kB activation, is prevalent. Intracellular GSH and Matrix metallopeptidase 9 (MMP-9) plasma levels parallel the transcription factors’ activation patterns and remain elevated throughout the observation time (24 h). This confirms that, in vivo, the return to normoxia after MH is sensed as a hypoxic trigger characterized by HIF-1 α activation. On the contrary, HH and VHH induce a shift toward an oxidative stress response, characterized by NRF2 and NF-kB activation in the first 24 h post-exposure.

1. Hypoxic Oxygen levels

To reach hypoxic oxygen levels, two different modalities are possible: one is dependent on a higher percentage of nitrogen in the inhaled mixture at atmospheric pressure (normobaric hypoxia or dilution hypoxia), while the other modality requires a reduced atmospheric pressure to reach lesser oxygen molecules per volume of breathed air (hypobaric hypoxia, such as that found during altitude stay or in a hypobaric chamber) [15].

Leveque et al. [16] compared the metabolic responses of normobaric hypoxic breathing for 1 h to inspired fractions of 10% and 15% oxygen in healthy humans (roughly mimicking altitudes of 6000 and 2400 m) [17]. Blood samples were taken before, and at 30 min, 2 h, 8 h, 24 h, and 48 h after exposure. The level of oxidative stress was evaluated by considering reactive oxygen species (ROS), nitric oxide metabolites (NOx), lipid peroxidation, and immune inflammation by interleukin-6 (IL-6) and neopterin, while antioxidant systems were observed in terms of the total antioxidant capacity (TAC) and urates. Hypoxia abruptly and rapidly increased ROS, while TAC showed a U-shape pattern, with a nadir between 30 min and 2 h. The regulation of ROS and NOx could be explained by the antioxidant action of uric acid and creatinine. The kinetics of ROS allowed for the stimulation of the immune system, as shown by increases in neopterin [18], IL-6, and NOx. This study provides insights into the mechanisms through which acute hypoxia affects various bodily functions and how the body sets up the protective mechanisms to maintain redox homeostasis in response to oxidative stress.

Another approach to acute hypoxia (12.5% of inspired fraction) was proposed by Mrakic-Sposta et al. [19]. Exposure to acute normobaric hypoxia elicited reactive oxygen species (ROS) accumulation [20], whose production kinetics and oxidative damage were investigated [21,22]. Subjects were monitored while breathing a hypoxic mixture (0.125 FiO_2_ in air, mimicking about 4100 m) and during recovery with room air. ROS production was assessed using Electron Paramagnetic Resonance in capillary blood. Total antioxidant capacity, lipid peroxidation (TBARS and 8-iso-PFG2alpha) [23,24], protein oxidation (PC), and DNA oxidation (8-OH-dG) were measured in plasma and/or urine [23,25]. The ROS production rate was monitored (5, 15, 30, 60, 120, 240, and 300 min). A production peak (+50%) was reached at 4 h. The on-transient kinetics, exponentially fitted (t(1/2) = 30 min r(2) = 0.995), were ascribable to the low O_2_ tension transition and the mirror-like related SpO(2) decrease: 15 min: −12%; 60 min: −18%. The exposure did not seem to affect the prooxidant/antioxidant balance. Significant increases in PC (+88%) and 8-OH-dG (+67%) at 4 h in TBARS (+33%) one hour after hypoxia offset were also observed. General malaise was described by most of the subjects. Under acute NH, ROS production and oxidative damage resulted in time and SpO_2_-dependent reversible phenomena.

In an animal model (mice), Shao et al. [26] studied the impact of hypoxia (a total of 30 mice were randomly divided into three groups (10 mice in each): control (CON) and chronic hypoxia (continuously with 13% O_2_ for 1 and 3 days (H1D and H3D), respectively) on advanced brain function (learning and memory skills in particular), and the effects of hypoxic stress on hippocampal function were assessed. Specifically, the effects of the dysfunction of mitochondrial oxidative phosphorylation using global proteomics. The authors found that hypoxic stress impaired cognitive and motor abilities, whereas it caused no substantial changes in the brain morphology or structure of mice. Bioinformatics analysis reported that hypoxia affected the expression of 516 proteins, of which 71.1% were upregulated and 28.5% were downregulated. The mitochondrial function was altered and manifested as a decrease in NADH dehydrogenase (ubiquinone) 1 alpha subcomplex 4 expression, accompanied by increased reactive oxygen species generation, resulting in further neuronal injury.

Their results may provide some new insights into how hypoxic stress alters hippocampal function via the dysfunction of mitochondrial oxidative phosphorylation [27].

2. Normobaric Hyperoxic Oxygen levels

In clinical practice, preventing or counteracting hypoxia is achieved providing normobaric oxygen. Even though hyperoxia may seem harmless, it can have detrimental effects even at modest levels if administered for prolonged periods, especially in critically ill patients [28].

One example is preterm babies, since their postnatal exposure to factors such as high oxygen concentrations may likely adversely influence postnatal growth and ongoing organ development [29,30,31,32]. The renal consequences of preterm births have attracted increasing attention and include a high risk of chronic kidney disease. The third trimester of pregnancy is the most active period of fetal nephrogenesis, during which more than 60% of nephrons are formed. Preterm birth (within <37 gestational weeks) interrupts the development and maturation of the kidneys during the critical growth period since neonates born preterm have an immature antioxidant defense system [33] and present an imbalance between the oxidant and the antioxidant system, leading to an increased level of oxygen free radicals, with subsequent increased risk of oxidative damage to organs.

Hyperoxia during the neonatal period impairs renal tubular development. Human and animal studies have demonstrated that neonatal hyperoxia increases oxidative stress and induces glomerular and tubular injuries, which are manifested as renal corpuscle enlargement, renal tubular necrosis, interstitial inflammation, and kidney fibrosis during the perinatal period [34,35,36].

Huang et al. [37] showed a global approach in their manuscript (see their Figure 1); they analyzed several animal studies (murine) of preterm birth interrupting the development and maturation of the kidneys during the critical growth period. They found that kidneys can exhibit structural defects and functional impairment due to hyperoxia. Furthermore, hyperoxia during nephrogenesis impairs renal tubular development and induces glomerular and tubular injuries, which manifest as renal corpuscle enlargement, renal tubular necrosis, interstitial inflammation, and kidney fibrosis. Preterm birth along with hyperoxia exposure induces a pathological predisposition to chronic kidney disease. Hyperoxia-induced kidney injuries are influenced by several molecular factors, including hypoxia-inducible factor-1 alpha and interleukin-6/Smad2/transforming growth factor-beta and Wnt/beta-catenin signaling pathways; these are key to cell proliferation, tissue inflammation, and cell membrane repair. Hyperoxia-induced oxidative stress is characterized by the attenuation or the induction of multiple molecular factors associated with kidney damage. This review focuses on the molecular pathways involved in the pathogenesis of hyperoxia-induced kidney injuries.

In terms of human studies on hyperoxia, Leveque et al. [38] compared two 60 min hyperoxic exposures on healthy humans. Since the effects of oxygen over time and at different partial pressures remain poorly understood, they measured the metabolic responses of a normobaric oxygen intake for 1 h to mild (30%) and high (100%) inspired fractions. Blood samples were taken before the intake, and at 30 min, 2 h, 8 h, 24 h, and 48 h after the single oxygen exposure. The level of oxidation was evaluated by the rate of reactive oxygen species (ROS) and the levels of isoprostane. Antioxidant reactions were observed by total antioxidant capacity (TAC), superoxide dismutase (SOD), and catalase (CAT). The inflammatory response was measured using interleukins-6 (IL-6), neopterin, creatinine, and urates. Oxidation markers increased from 30 min on to reach a peak at 8 h. From 8 h post-exposure, the markers of inflammation increased more significantly in the 100% condition than in the 30% condition. This study suggests a biphasic response over time characterized by an initial “permissive oxidation” followed by increased inflammation and the antioxidant protection system seems to not be the leading actor. The authors concluded that the kinetics of enzymatic reactions need to be better studied to establish therapeutic, training, or rehabilitation protocols for a more targeted use of oxygen.

3. Hyperbaric Hyperoxic Oxygen levels

To reach oxygen levels above 100%, a hyperbaric environment is needed; increasing the surrounding pressure is achieved in a hyperbaric chamber and is usually achieved for therapeutic reasons. Hyperbaric oxygen therapy (HBOT) is a therapeutic approach based on breathing pure oxygen (O_2_) in an augmented atmospheric pressure [39,40].

Two directions are found in the field of immature (or premature) organisms; on the one hand, as previously reported, some adverse normobaric hyperoxic effects may occur, but on the other hand, several sessions of hyperbaric oxygen treatment have been reported to be beneficial [29,41,42].

In a murine model, Jeremic et al. [43] showed evidence suggesting that hyperbaric oxygenation may affect the activity of adult neural stem cells (NSCs) [44,45,46]. Since the role of NSCs in recovery from brain injury is still unclear, the purpose of this study was to investigate the effects of sensorimotor cortex ablation (SCA) and HBO treatment (HBOT) on the processes of neurogenesis in the adult dentate gyrus (DG), a region of the hippocampus that is the site of adult neurogenesis. Ten-week-old Wistar rats were divided into groups: Control (C, intact animals), Sham control (S, animals that underwent the surgical procedure without opening the skull), SCA (animals in whom the right sensorimotor cortex was removed via suction ablation), and SCA + HBO (operated animals that passed HBOT). HBOT protocol: pressure applied at 2.5 absolute atmospheres for 60 min, once daily for 10 days. Using immunohistochemistry and double immunofluorescence labeling, it was shown that SCA causes significant loss of neurons in the DG. Newborn neurons in the subgranular zone (SGZ), inner third, and partially mid-third of the granule cell layer are predominantly affected by SCA. HBOT decreases the SCA-caused loss of immature neurons, prevents reduction of dendritic arborization, and increases the proliferation of progenitor cells.

A protective effect of HBO can be considered by reducing the vulnerability of immature neurons in the adult DG to SCA injury.

The only other way to increase oxygen partial pressure above one atmosphere is to breathe underwater while diving. A particular diving procedure using closed-circuit rebreathers (CCR) allows a constant PO_2_ to be set that will be kept stable during the whole dive regardless of depth variations (with concomitant ambient pressure changes). Arya et al. [47] measured blood-borne extracellular vesicles and inflammatory mediators in divers using closed-circuit rebreathing apparatus and custom-mixed gases to diminish some diving risks. “Deep” divers (n = 8) dove once to mean (±SD) 102.5 ± 1.2 m of sea water (msw) for 167.3 ± 11.5 min. “Shallow” divers (n = 6) dove three times on day 1 and then repeatedly over 7 days to 16.4 ± 3.7 msw for 49.9 ± 11.9 min. There were statistically significant elevations of microparticles (MPs) in deep divers (day 1) and shallow divers at day 7 that expressed proteins specific to microglia, neutrophils, platelets and endothelial cells, as well as thrombospondin (TSP)-1 and filamentous (F-) actin. Intra-MP IL-1β increased by 7.5-fold (*p* < 0.001) after day 1 and 41-fold (*p* = 0.003) at day 7. Intra-MP nitric oxide synthase-2 (NOS2) increased 17-fold (*p* < 0.001) after day 1 and 19-fold (*p* = 0.002) at day 7. Plasma gelsolin (pGSN) level decreased by 73% (*p* < 0.001) in deep divers (day 1) and 37% in shallow divers by day 7. Plasma samples containing exosomes and other lipophilic particles increased from 186 to 490% among the divers but contained no IL-1β or NOS2.

The authors concluded that diving triggers inflammatory events even when controlling for hyperoxia and many are not proportional to the depth of diving.

In terms of the therapeutic side of hyperbaric oxygen, it is strange that, despite having been used for years, the exact kinetics of the reactive oxygen species between different levels of hyperbaric oxygen exposure are still not clearly evidenced and, without much scientific evidence, it is common practice to apply HBOT sessions every 24 h [48]. The need for several sessions to reach a relevant effect is likewise commonly accepted, however, the optimal hyperbaric oxygen levels and the time needed between each session to optimize cellular responses—such as Hypoxia-inducible factor (HIF-1α) or nuclear factor kappa β (NF-Kβ), erythroid related factor 2 (NRF2), cellular vesicles, and microparticles, such as Caspase 3 [5,9,14]—are still debated and stand solely on observational clinical outcomes.

Leveque et al. [49] studied the metabolic responses of hyperbaric hyperoxia exposures for 1 h at 1.4 and 2.5 ATA.

Fourteen healthy non-smoking subjects volunteered for the study. Blood samples were taken before, and at 30 min, 2 h, 24 h, and 48 h after 1 h hyperbaric hyperoxic exposure. The level of oxidation was evaluated by the rate of ROS production, nitric oxide metabolites (NOx), and the levels of isoprostane. Antioxidant reactions were assessed through measuring superoxide dismutase (SOD), catalase (CAT), cysteinylglycine, and glutathione (GSH). The inflammatory response was measured using interleukine-6, neopterin, and creatinine. A short (60 min) period of mild (1.4 ATA) and high (2.5 ATA) hyperbaric hyperoxia led to a similar significant increase in the production of ROS and antioxidant reactions. Immunomodulation and inflammatory responses, on the contrary, responded proportionally to the hyperbaric oxygen dose. Further research is warranted on the dose and the inter-dose recovery time to optimize the potential therapeutic benefits of this promising intervention.

These encouraging but also challenging results lead us to question if some oxygen levels formerly considered as ‘HBOT sham’ [50,51] may be of therapeutic interest [9,51,52].

Oxygen and its variations are certainly the most powerful cellular triggers that can be found in nature.

Returning to Philippus Theophrastus Aureolus Bombast von Hohenheim (Paracelsus), he was known to have quite a hot temper and be “bombastic”. Nevertheless, he was very respectful of previous scholars, such as Aulus Cornelius Celsus (who wrote “De Medicina”); he respected deep knowledge, which is likely why the name “Paracelsus” was appropriate to him, since he considered himself not equal to Celsus.

Oxygen can be considered bombastic sometimes, but is also as indispensable; deeper knowledge on its biology is vital in the present day, and the simple “dose is the poison” approach is not appropriate for such a metabolically active molecule [41].

To better describe this concept, we constructed Figure 2 after three manuscripts from Leveque et al. [16,38,49] to illustrate how relative changes of reactive oxygen species are not directly dependent on the dose up to 48 h following 60 min of exposure.

As can be seen from Figure 2, no real difference in ROS production is present during hyperoxia for as long as 48 h between two rather different doses of oxygen on the hypoxic side; on the contrary, some acute differences even in slightly different doses can be seen (in the first few hours after exposure). The dose seems to not be the only clue!

This editorial, drawing a general picture of the Special Issue it accompanies, clearly shows how much new material can aid understanding of the underlying mechanisms elicited by oxygen exposures.

Oxygen biology is still a very fruitful research field, and we strongly believe that “oxygen variations” will, in the coming years, be a promising and proficuous field to explore.

## Figures and Tables

**Figure 1:**
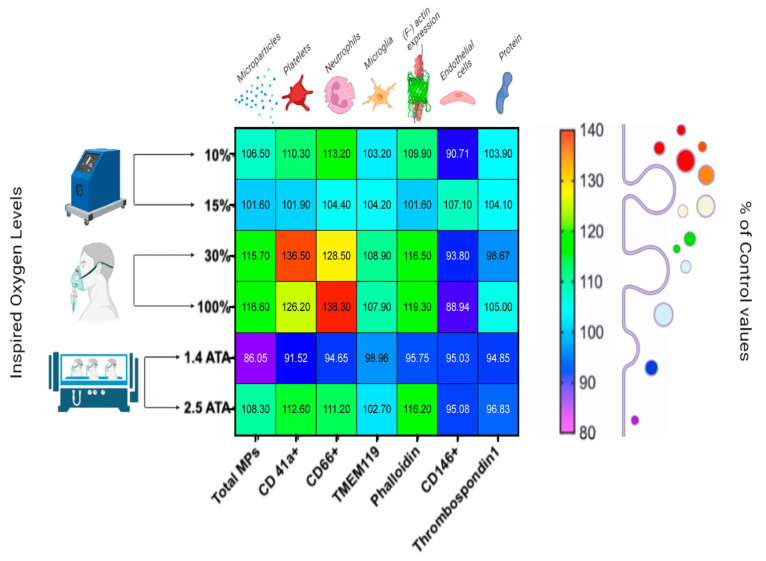
Percentual variations in microparticles (MPs) after 60 min of oxygen breathing. Levels of oxygen are shown on the ordinate, and total MPs and MP sub-types are shown on the abscissa. Blood sampling occurred 120 min after exposures (in a total of 48 subjects). Results are expressed in the heat map as mean percentage change (modified from [9]).

**Figure 2:**
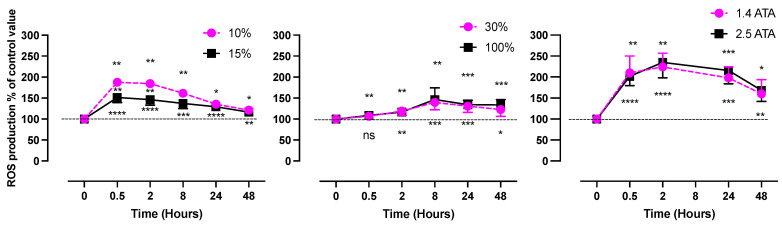
Percentual variations in ROS production after 60 min of oxygen breathing. Levels of oxygen are shown in the figure legend (48 subjects in total). Results are expressed as mean percentage change of control values (modified from [16,38,49]) (*: *p* < 0.05, **: *p* < 0.01, ***: *p* < 0.001, ****: *p* < 0.0001; RM-ANOVA and Dunnet’s post hoc test).
